# Synchrotron Radiation X-Ray Phase-Contrast Tomography Visualizes Microvasculature Changes in Mice Brains after Ischemic Injury

**DOI:** 10.1155/2016/3258494

**Published:** 2016-07-31

**Authors:** Peng Miao, Zhixia Wu, Miao Li, Yuanyuan Ji, Bohua Xie, Xiaojie Lin, Guo-Yuan Yang

**Affiliations:** ^1^School of Communication and Information Engineering, Shanghai University, Shanghai 200444, China; ^2^Med-X Research Institute, Shanghai Jiao Tong University, Shanghai 200030, China

## Abstract

Imaging brain microvasculature is important in plasticity studies of cerebrovascular diseases. Applying contrast agents, traditional *μ*CT and *μ*MRI methods gain imaging contrast for vasculature. The aim of this study is to develop a synchrotron radiation X-ray inline phase-contrast tomography (SRXPCT) method for imaging the intact mouse brain (micro)vasculature in high resolution (~3.7 *μ*m) without contrast agent. A specific preparation protocol was proposed to enhance the phase contrast of brain vasculature by using density difference over gas-tissue interface. The CT imaging system was developed and optimized to obtain 3D brain vasculature of adult male C57BL/6 mice. The SRXPCT method was further applied to investigate the microvasculature changes in mouse brains (*n* = 14) after 14-day reperfusion from transient middle cerebral artery occlusion (tMCAO). 3D reconstructions of brain microvasculature demonstrated that the branching radius ratio (post- to preinjury) of small vessels (radius < 7.4 *μ*m) in the injury group was significantly smaller than that in the sham group (*p* < 0.05). This result revealed the active angiogenesis in the recovery brain after stroke. As a high-resolution and contrast-agent-free method, the SRXPCT method demonstrates higher potential in investigations of functional plasticity in cerebrovascular diseases.

## 1. Introduction

Rodent models have been widely used in plasticity studies of cerebrovascular diseases, like arteriovenous malformation, ischemic or hemorrhagic stroke, and vasospasm. Obtaining intact brain vasculature is a prerequisite for further understanding of cerebrovascular disorders. Conventional X-Ray Angiography, Magnetic Resonance Angiography (MRA), and Positron Emission Tomography (PET) can provide vasculature information in human and animals but with limited spatial resolutions [1–7]. Optical imaging methods (confocal or two-photon microscope) provide sufficient spatial resolution, but the penetration depth is limited for deep brain applications [8, 9]. The vascular corrosion casting method allows more detailed examination of vessel morphology including some but not all of the microvasculature in the brain [10, 11]. Recently, *μ*CT [12] and *μ*MRI [13] were developed to obtain high-resolution (~30 *μ*m) brain vasculature in small animals* ex vivo* using contrast agents. All the above methods need contrast agents to enhance the imaging contrast of vasculature, which may result in bad reconstruction under inadequate perfusion of contrast agents. Furthermore, contrast agents cannot be applied in brain with hemorrhagic symptom. Therefore, investigations of cerebrovascular diseases need a contrast-agent-free vasculature imaging method with sufficient penetration depth and imaging resolution.

In X-ray imaging, besides the absorption contrast, the phase contrast [14, 15] has also been utilized in imaging biological tissues [16]. Generally, X-ray PCI provides the cross section of phase shift ~1000 times higher than that of absorption. For example, in* in vivo* imaging of lungs and sinuses, air-tissue interface provides sufficient phase contrast in X-ray phase-contrast imaging (PCI) [17, 18]. Furthermore, gas-tissue interface also provides better phase contrast than blood-tissue interface and therefore has been used in imaging the anatomy of kidney [19], ear [20], and brain [21]. Previous studies showed that the type of gas did not affect the image contrast [22]; thus, both CO_2_ gas and air can be used. In the morphological studies of brain microvasculature, the gas-tissue interface may also be utilized for high-resolution label-free imaging. But adequate preparation method for brain sample needs to be developed to maintain the original morphology of intact brain vasculature and at the same time create stable air-tissue interface.

Traditional X-ray PCI uses interferometry and crystal analyzer to obtain the phase information [23]. Without interferometry, inline X-ray PCI method can also retrieve the phase information and is used in a variety of studies [24–26]. Synchrotron radiation (SR), with its higher intensity, better coherence, and smaller divergence, is an ideal source for X-ray PCI. It can greatly improve the imaging resolution and sensitivity for brain vasculature imaging. Previous studies have already shown the imaging potentials of synchrotron radiation for brain vasculature [27–29]. Here, in this study, we focused on the phase contrast and high resolution which improved the ability of synchrotron radiation imaging in mouse brain vasculature. We developed an inline synchrotron radiation X-ray phase-contrast tomography (SRXPCT) method for* in vitro* brain vasculature imaging with high resolution. The SRXPCT imaging setup was constructed and optimized at the BL13W1 beamline of Shanghai Synchrotron Radiation Facility (SSRF). A specific sample preparation protocol was proposed to maintain the intact brain vasculature and achieve high phase contrast of vasculature using air-tissue interface. Finally, the proposed SRXPCT method was applied to investigate the microvasculature changes in mouse brain after ischemic injury from transient middle cerebral artery occlusion (tMCAO) model.

## 2. Materials and Methods

### 2.1. Imaging Setup for SRXPCT

**Figure 1:**
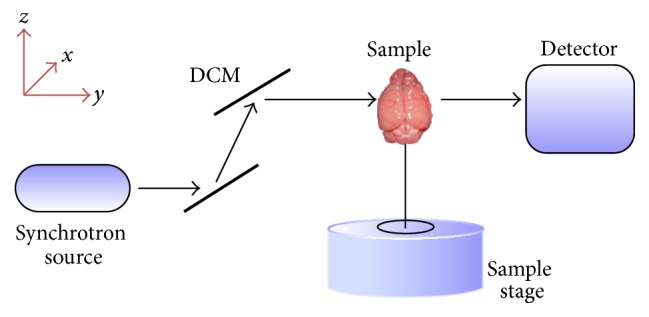
Schematic of the imaging system of SRXPCT. The monochromatic SR system at the BL13W1 beamline outputs 16 KeV X-ray illuminating the brain sample. The sample was rotated on the sample stage for CT reconstruction. Images were recorded by a high-resolution CCD (pco. 2000, PCO AG, Kelheim, Germany) with 1 : 2 microscopic lens and Yag:Ce scintillator.

**Figure 2:**
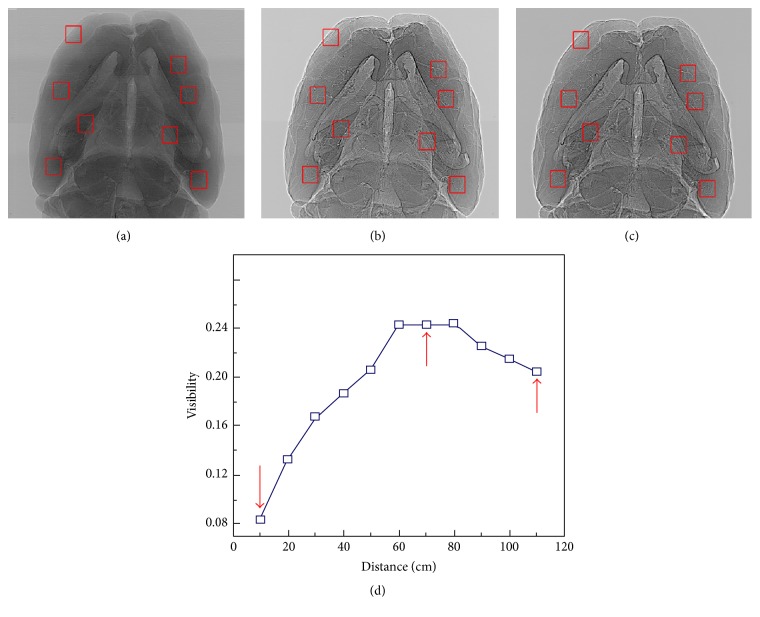
Optimization of SRXPCT: corrected phase images acquired with different sample-to-detector distances ((a) 10 cm, (b) 70 cm, and (c) 110 cm). (d) The averaged visibilities of all boxed areas in (a)–(c) at different sample-to-detector distances.

**Figure 3:**
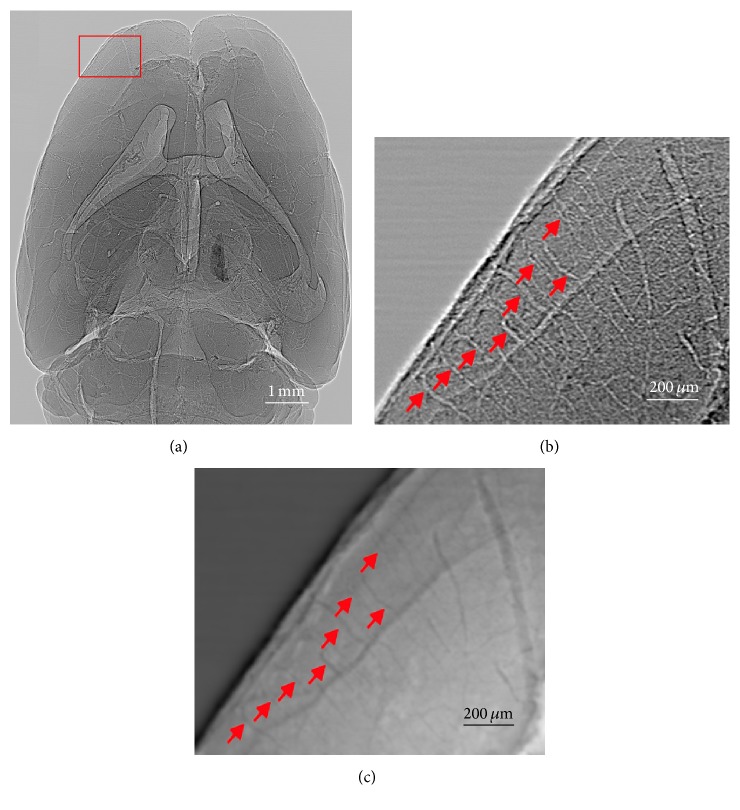
The corrected phase image of entire mouse brain sample (in air) based on the proposed method. Both cerebral arteries and veins were revealed in (a). The brain structures can also be identified including cerebral cortex, the lateral ventricle (LV), and the third ventricle of cerebrum thalamus and cerebellum (CB). (b) Enlarged image of red box area in (a). (c) The phase retrieved image of (b).

The imaging setup (Figure 1) of SRXPCT was constructed at the BL13W1 beamline in SSRF. The energy range of outputting X-ray was from 8 KeV to 72.5 KeV and its beam width was up to 10 mm × 10 mm (horizontal × vertical) with divergence of 1.5 mrad × 0.2 mrad (horizontal × vertical). The setup of our SRXPCT included the monochromators for 16 KeV, a specimen table, and a high-resolution CCD recording system. Silicon double crystal monochromator (DCM) was used to obtain parallel 16 KeV X-ray beam. The distance from light source to the specimen table was fixed at 34 meters. The distance from sample to detector was optimized before the imaging experiments (Figure 2). A microscope with 1 : 2 lens coupled to a 100 *μ*m Yag:Ce scintillator was used in the imaging system. And a CCD camera (pco. 2000, PCO AG, Kelheim, Germany) with pixel size of 7.4 *μ*m × 7.4 *μ*m was used to record the original phase images *I*(*x*, *y*) (2048 × 2048 pixels). The overall effective pixel size in the recorded image was 3.7 *μ*m × 3.7 *μ*m. The exposure time for high-resolution imaging was 200 ms. Dark-field image *I*_*d*_(*x*, *y*) and flat-field image *I*_*f*_(*x*, *y*) were collected before sample imaging and used in preprocessing (flat-field and dark-field corrections) to obtain the corrected phase image *I*_*p*_(*x*, *y*) (1). In the corrected phase images *I*_*p*_(*x*, *y*), brain vasculature was enhanced (Figures 2(a), 2(b), 2(c), 3(a), and 3(b)). Hence,

(1) $$\begin{array}{c} I_{p}\left(\;x,y\;\right)=\frac{I\left(x,y\right)-I_{d}\left(x,y\right)}{I_{f}\left(x,y\right)-I_{d}\left(x,y\right)}. \end{array}$$

The phase retrieved image (Figure 3(c)) can be calculated from the corrected phase image *I*_*p*_(*x*, *y*) using Paganin et al.'s method [30, 31]. Although the phase retrieved image presented the normal brain anatomy, the imaging contrast of vasculature was very limited especially for investigations of microvasculature. Therefore, in this study, we proposed to use the corrected phase images *I*_*p*_(*x*, *y*) instead of the phase retrieved images to obtain CT reconstructions of brain vasculature.

### 2.2. Optimization of SRXPCT

To optimize the vasculature imaging performance, different sample-to-detector distances (10 cm~110 cm) were tested and compared. The visibility parameter *V* of different areas (rectangles indicated in Figures 2(a)–2(c)) in the corrected phase images with different sample-to-detector distances was computed (2). In Figure 2(d), with the range from 10 cm to 80 cm, the vasculature was distinguishable at 30 cm and became clearer as the distance increased. But there was no significant improvement on the imaging quality from 60 cm to 80 cm. So, the optimal sample-to-detector distance for our setup was in the range of 60 cm~80 cm. Hence,

(2) $$\begin{array}{c} V=\frac{\left(I_{max}-I_{min}\right)}{\left(I_{max}+I_{min}\right)}, \end{array}$$

where *V* is the visibility parameter and *I*_max_ and *I*_min_ are the maximum and minimum values in the selected area in corrected phase image.

### 2.3. CT Reconstruction

For CT reconstruction, the sample stage rotates by 180° with 0.12° interval and a total of 1500 projections were obtained with 2048 × 2048 pixel size and 16-bit dynamic range. The brain samples were embedded in an Eppendorf tube using agarose gel. The tube was then directly fixed on the sample stage to keep it stationary in rotating. The tomogram had a total exposure time of 5 min (200 ms exposure time for each projection) giving a radiation dose of about 2 Gy. CT slices of brain samples were reconstructed directly based on the corrected phase images using a modified filtered back projection reconstruction algorithm [32] written by Interactive Data Language (IDL 7.0). Brain vasculature was then segmented based on the 3D extension of ridge based method [33] by Matlab 7.10.0 (MathWorks, Inc., Miami, FL). In reconstructed 3D CT data, ridges were detected based on local maxima of intensities. And vasculature was modelled as asymmetric Gaussian functions whose parameters constituted ridge descriptors. Then, this local information is used to cluster the ridges, which leads to the final threshold and segmentation of brain vasculature. ImageJ software (NIH, USA) was used for 3D volume rendering of whole or part of the brain vasculature.

### 2.4. Animal Model of tMCAO

Adult male C57BL/6 mice (*n* = 14) weighing 25~30 grams were randomly selected as sham group (*n* = 7) and brain injury group (*n* = 7). The experimental protocol used in this study was approved by the Institutional Animal Care and Use Committee of Shanghai Jiao Tong University. Transient middle cerebral artery occlusion (tMCAO) was performed based on our previous study [34]. In the surgery, mice were anesthetized with ketamine/xylazine (100 mg/10 mg/kg, Sigma) intraperitoneally. Body temperature was maintained at 37 ± 0.3°C using a heating pad (RWD Life Science, Shenzhen, China). After isolation of the left common carotid artery (CCA) and external and internal carotid arteries (ECA and ICA), a silicone-coated 6-0 suture (Covidien, Mansfield, MA) was gently inserted from the ECA stump to the ICA and stopped at the opening of the middle cerebral artery (MCA). The distance from the bifurcation of ICA/ECA to MCA was about 10 ± 0.5 mm. Successful occlusion was confirmed by Laser Doppler Flowmetry (Moor Instruments, Devon, UK). Reperfusion started 90 min after MCAO with suture withdrawal. Mice in the sham group underwent the same procedure except for inserting the suture into the ICA. After 14 days of reperfusion [35], all mice were prepared for the SRXPCT imaging of brain vasculature.

### 2.5. Preparation of Brain Sample

On the 14th day after reperfusion, mice were anesthetized again using ketamine/xylazine (100 mg/10 mg/kg, Sigma) intraperitoneally. Under anesthesia, thoracotomy was performed and a blunt needle was gently inserted into the left ventricle of beating heart. The needle was then connected with a three-way valve, which was further connected with a normal saline container and 4% paraformaldehyde (PFA) container. 0.9% normal saline (37°C) was perfused by the beating heart in the vasculature system at 100 mmHg until the outflow from the right atrium was colorless. At the end of the normal saline perfusion, the valve was switched to the 4% PFA buffer to fix the brain vessel walls. Then, animals were sacrificed and brain samples were removed and fixed in PFA buffer overnight at 4°C. The fixed brains were then dehydrated with 100% ethyl alcohol for 24 hours to keep the sample morphology normal. Finally, ethyl alcohol evaporated and air occupied the intact brain vasculature by the 24-hour dry procedure. After sample preparation, all brain samples were imaged by our SRXPCT system and high-resolution 3D vasculature was reconstructed.

## 3. Results

### 3.1. Imaging Performance

Figure 3(a) showed one corrected phase image of the entire normal mouse brain obtained by SRXPCT. Different brain structures including cerebral cortex, ventricle, basal ganglia, thalamus, and cerebellum can be identified in the image. The contrast of brain vasculature was sufficiently high for blood vessel extractions. Figures 3(b) and 3(c) demonstrated the enlarged phase-contrast image (Figure 3(b)) and its phase retrieved image (Figure 3(c)) of the cerebral cortex area (red rectangle in Figure 3(a)) where the penetrating arterioles (red arrows in Figures 3(b) and 3(c)) branching from cortical arteries can be easily identified. The smallest diameter of distinguishable vessel was about 7.4 *μ*m.

### 3.2. CT Reconstruction

**Figure 4:**
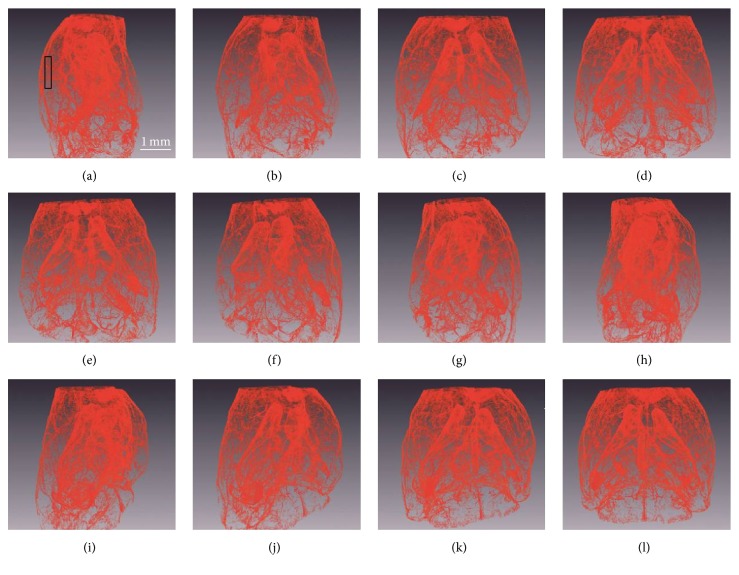
3D reconstruction of entire mouse brain vasculature. The rendered vasculature is rotated by 15° in each image ((a) to (l)). The scale bar is 1 mm.

**Figure 5:**
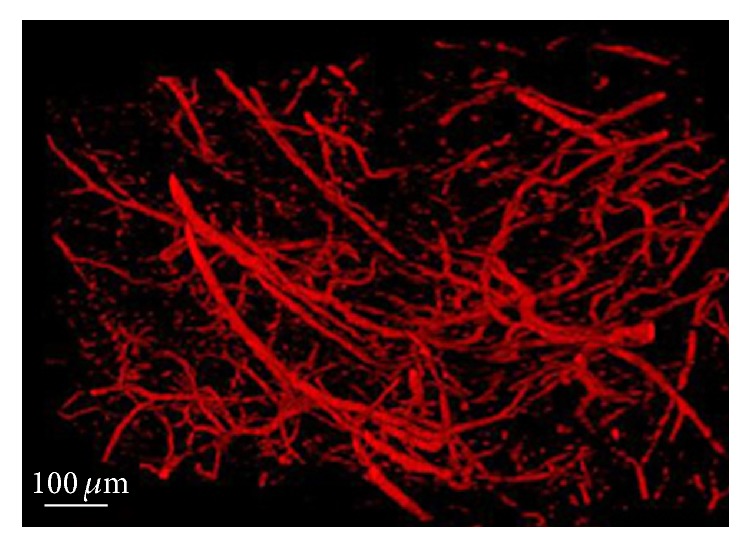
Enlarged 3D view of microvasculature in the brain region of interest (black box area in Figure 4(a); also see the supplementary video, in Supplementary Material available online at http://dx.doi.org/10.1155/2016/3258494). The scale bar is 100 *μ*m.

After CT reconstruction and vasculature segmentation, 3D vasculature of the entire mouse brain was obtained. Figures 4(a)–4(l) presented volume rendering of vasculature at different viewpoints (15° rotation between images). The whole view of vasculature was useful in visualization of large vessels. In studies of microvasculature, 3D view of the brain region of interest was more suitable for analysis (Figure 5 and supplementary video).

**Figure 6:**
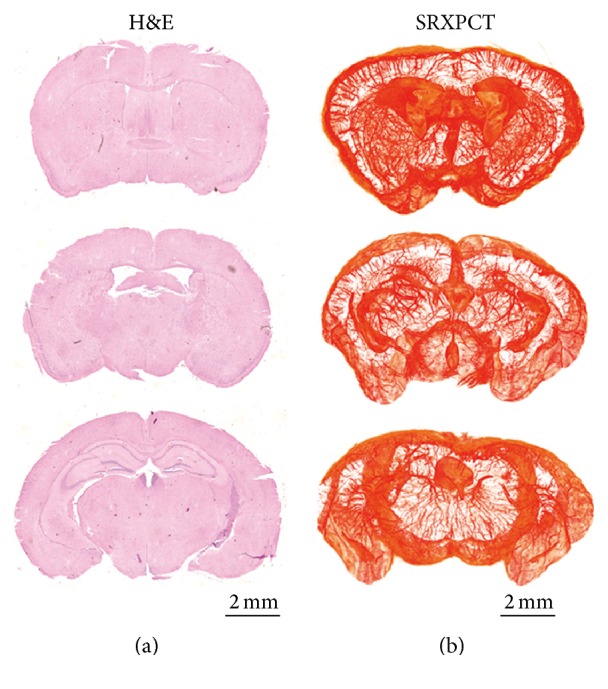
Comparison of H&E staining (a) and reconstructed sections of 3D SRXPCT (b) of mice brains.

As a nondestructive method, SRXPCT can provide 3D re-sections of brain anatomy with detailed vasculature network (Figure 6). For studies of cerebrovascular disease, these 3D re-sections are better visualization than histological slices based on traditional methods like H&E staining (Figure 6).

### 3.3. Changes of Microvasculature in Mouse Brain after Ischemic Injury

In this study, the proposed SRXPCT method was applied to investigate the microvasculature changes in mouse brain after ischemic injury from tMCAO model. After 90 min occlusion of MCA in injury group, mouse brain was reperfused for 14 days. On the 14th day after surgery, all mouse brains in both injury group and sham group were prepared for SRXPCT.

Based on the segmentation of 3D vasculature, both vessel diameters and branching points were collected. After 14 days of reperfusion, the mice brains recovered from ischemic injury with angiogenesis and microvasculature changes [35]. To investigate this vasculature change, each branching point in the segmented vasculature was identified and the prebranching and postbranching vessel radii were calculated. Since, for one branching point, the postbranching vessels may have different vessel radius, we use the averaged radius as the postbranching radius. Because we were interested in the microvasculature changes, only small branching vasculature (with prebranching radius < 20 *μ*m) was summarized and analyzed in the next step. We define a parameter, the radius ratio *K* (defined as the ratio of postbranching vessel radius to prebranching vessel), to quantitatively analyze the microvasculature changes.

**Figure 7:**
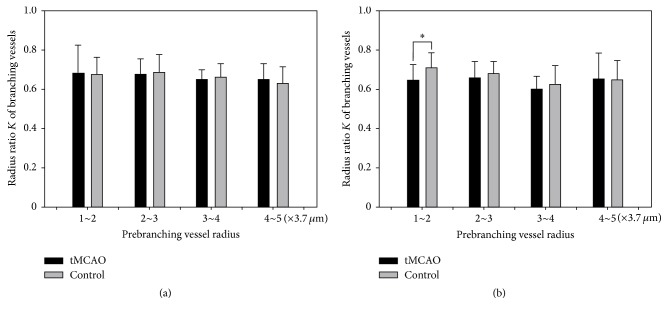
Microvasculature changes in contralateral (a) and ipsilateral (b) brains after 14-day reperfusion from tMCAO model. In ipsilateral brain, for the branching vessels with radius < 7.4 *μ*m, brain injury group demonstrated a statistically significant change in vessel radius ratio *K* (defined as the radius ratio of postbranching vessel to prebranching vessel) compared with that in the sham group.  ^*∗*^Student's *t*-test *p* < 0.05.

Figure 7 showed the radius ratio *K* in contralateral (a) and ipsilateral (b) brains after 14 days of reperfusion from tMCAO model. In the contralateral brain without injury, there were no statistically significant differences in *K* between sham and injury groups (Figure 7(a)). However, for the ipsilateral brain (injury side) in injury group, the radius ratio *K* of vessels with radius < 7.4 *μ*m was significantly smaller than that in the sham group (Figure 7(b)). Meanwhile, larger branching vessels (radius > 7.4 *μ*m) demonstrated no statistically significant differences between the two groups (Figure 7(b)). Because active angiogenesis in the recovery from stroke leads to microvasculature rebuilding [35], newborn small branching vessels can decrease the radius ratio *K* which was consistent with our statistical result. The 3D rendering further confirmed that peri-infarct areas were the main sites of reduced radius ratio *K* corresponding to angiogenesis, which was also consistent with previous studies [36].

## 4. Discussions

In this study, we developed SRXPCT method for* in vitro* high-resolution 3D imaging of intact rodent brain (micro)vasculature. Using the specific preparation of brain sample, the phase contrast from density difference over air-tissue interface was obtained in intact brain vasculature. Applying the new method, there was no need of X-ray contrast agent to obtain microvasculature changes in studies of cerebrovascular diseases.

X-ray phase-contrast imaging method obtained high imaging contrast from the phase change of X-rays once they cross the interfaces with inhomogeneous refractive indices. Studies indicated that the lung was well suited to be characterized by X-ray phase-contrast imaging because it was comprised approximately of 80% of air at end expiration, divided by thin tissue structures [17, 18]. The brain vasculature had no air-tissue interface* in vivo*. Another study showed that the phase contrast can be utilized for anatomy imaging in the* in vitro* sample without fixation (biological fresh sample) [37]. This strategy also failed for brain vasculature imaging. The X-ray wave fronts were refracted at each interface between blood flow (in vessel lumen) and surrounding tissue. It was difficult to enhance the imaging contrast of vasculature in* in vivo* status or in* in vitro* status without fixation. At the same time, potential applications of air-tissue interface for brain vasculature can be utilized in* in vitro* imaging strategy. However, proper techniques for the perfusion, fixation, and dehydration of brain sample were crucial to maintain the integrity and original morphology of brain vasculature as well as achieve the phase contrast of air-tissue interface.

About the resolution of SRXPCT, the pixel resolution of imaging is 3.7 *μ*m × 3.7 *μ*m, allowing the identification of brain microvasculature under 10 *μ*m. In our study, different levels of vasculature were well visualized. Although the vessels with radius of one-/two-pixel width can be detected in the current study, higher resolution imaging system is highly suggested and may provide more details of microvasculature even the capillary network. Better understanding of brain vessel architecture and vessel alterations would be beneficial for investigations of brain diseases [10]. Compared to *μ*CT method, SRXPCT does not require the usage of contrast agent. In addition, SRXPCT can also provide morphology information of both brain vasculature and the ventricles and nuclei.

There are also certain limitations associated with the current SRXPCT method. The casting method can be applied as the gold standard for optimization of imaging quality which has not been applied in this study. Although the resolution of PCI images is high enough to observe vessels with diameters less than 10 *μ*m, it is still challenging to acquire detailed angiogenesis procedure like blood vessel sprouting. Also, additional experiments need to be performed to obtain the continuous observations of microvasculature changes after stroke. At the current stage, SRXPCT can be used only for specifically prepared mouse brain samples. For larger samples, like the rat brain, the current SRXPCT technique can also work but needs larger FOV camera and longer preparation time for sample fixation and dehydration. There are big challenges in* in vivo* SRXPCT, because no current phase-contrast mechanisms can be used in the* in vivo* applications.

## Supplementary Material

The supplementary video demonstrated the 3D view of microvasculature in the brain region corresponding to Figure 5.
